# When Local Bone Pain Is Just the Tip of the Iceberg—A Case Report of Three Patients With Chronic Multifocal Recurrent Osteomyelitis and Some Red Flags to Help Make the Diagnosis

**DOI:** 10.3389/fped.2019.00407

**Published:** 2019-10-14

**Authors:** Holly Wobma, Diego Jaramillo, Lisa Imundo

**Affiliations:** ^1^Adolescent Rheumatology, Columbia University Medical Center, New York, NY, United States; ^2^Division of Pediatric Radiology, Morgan Stanley Children's Hospital, New York, NY, United States

**Keywords:** chronic non-bacterial osteomyelitis, pustulosis, inflammatory bowel disease, pediatric, bone pain

## Abstract

Chronic recurrent multifocal osteomyelitis (CRMO) is an uncommon cause of chronic inflammatory bone pain in children that can be disabling. Often, this diagnosis is considered only after a prolonged workup, leading to frustration for families and unnecessary interventions for patients. Here we describe three cases of CRMO to increase awareness of how it may present. The first patient had a typical presentation of focal bone pain (knee), for which she underwent bone scan (hint of >1 lesion), had a bone biopsy to rule out malignancy, received empiric antibiotics for presumed infection, and finally had whole-body imaging confirming CRMO when symptoms persisted. The second patient had a similar workup, but initially presented with clavicular pain. This location should raise suspicion for CRMO, as it is an uncommon location for infectious osteomyelitis. The third patient presented with delayed growth and right hip pain, and simultaneously developed palmoplantar pustulosis. These secondary findings can also serve as red flags for CRMO, as it has been linked to this skin condition and inflammatory bowel disease. All patients improved on non-steroidal anti-inflammatory (NSAID) medications, methotrexate, and/or tumor necrosis factor (TNF)-α antagonists. By raising awareness of clinical findings suggestive of CRMO, this report may help expedite diagnosis, so patients can be started on anti-inflammatory therapy.

## Introduction

Chronic recurrent multifocal osteomyelitis (CRMO) is an auto-inflammatory disease in children with a reported incidence of 0.4 per 100,000 (1). Most patients have a disease onset between 7 and 12 years of age, and girls are disproportionately affected (2). Clinical presentation involves at least one region of focal bone pain that may cause functional impairment. Symptoms may relapse and remit, and the affected region can migrate between one flare and the next (3). Often full body imaging will reveal ≥4 lesions of osteomyelitis, particularly in the lower extremities, vertebrae, and clavicles (4, 5). CRMO is also affiliated with other inflammatory processes, particularly inflammatory bowel disease (IBD), spondylitis, psoriasis, and palmoplantar pustulosis (2, 6). The most significant clinical consequences for children include chronic pain and disability, leg length discrepancy (if lesions involve the growth plates), and failure to thrive (if associated with IBD) (7, 8). Once started on anti-inflammatory medications, the disease is often self-limited and usually resolves after a few years (3).

There are several diagnostic challenges for CRMO, which all inter-relate. First, even if a patient has multiple bone lesions, not all will have related symptoms of pain or tenderness. In addition, even in affected symptomatic areas, the lesions are often undetectable on plain radiographs. Whole body MRI (WBMRI) is the standard diagnostic modality, because it can reveal the multifocal, often symmetric nature of the disease and symptomatic and asymptomatic areas of involvement. However, when there is only one region of pain on presentation, the differential diagnosis in children is quite wide, including many conditions that are far more common and better known than CRMO (Table 1). Thus, it is not always obvious that a WBMRI is warranted. As a final challenge, there are no universally accepted diagnostic criteria for CRMO, although some have been suggested (Tables 2, 3) (9, 10). Altogether, these challenges mean that diagnosis often entails an extensive and prolonged workup by multiple specialists, and children may have been exposed to prolonged courses of antibiotics, which can lead to adverse effects and antimicrobial resistance. There are likely many cases that have never been diagnosed (7, 9).

**Table 1:** Differential diagnosis for presentation of bone pain ± fever.

**Infectious**	**Malignant**	**Traumatic**	**Auto-immune/inflammatory**	**Other**
- Bacterial osteomyelitis - Fungal osteomyelitis (coccidioidomycosis, cryptococcosis) - Brucellosis - Pott's Disease *(M. tuberculosis)*	- Osteoid osteoma - Osteosarcoma - Ewing sarcoma - Leukemia - Lymphoma - Langerhans cell histiocytosis - Skeletal metastases	- Bone bruise - Fracture - Stress injury	- CRMO - Juvenile Idiopathic Arthritis	- Gaucher's Disease - Sickle cell disease - Avascular necrosis

**Table 2:** Jansson criteria for diagnosis of CRMO.

**Major diagnostic criteria**	**Minor diagnostic criteria**
1. Radiologically proven osteolytic/-sclerotic bone lesion 2. Multifocal bone lesions 3. Palmoplantar pustulosis (PPP) or psoriasis 4. Sterile bone biopsy with signs of inflammation and/or fibrosis, sclerosis	A. Normal blood count and good general state of health B. CRP and ESR mildly-to-moderately elevated C. Observation time >6 months D. Hyperostosis E. Associated with other autoimmune diseases apart from PPP or psoriasis F. Grade I or II relatives with autoimmune or autoinflammatory disease

*Non-bacterial osteomyelitis (i.e., CRMO) is confirmed by 2 major or 1 major + 3 minor criteria*.

**Table 3:** Bristol Criteria for diagnosis of CRMO.

**The presence of typical clinical findings** (bone pain ± localized swelling without significant local or systemic features of inflammation or infection)	
AND	
**The presence of typical radiological findings** (plain x-ray: showing combination of lytic areas, sclerosis and new bone formation, or preferably STIR MRI: showing bone marrow edema ± bone expansion, lytic areas, and periosteal reaction)	
AND EITDER	
Criterion 1: >1 bone (or clavicle alone) without significantly raised CRP (<30 mg/L)	Criterion 2: if unifocal disease (other than clavicle), or CRP >30 mg/L, with bone biopsy showing inflammatory changes (plasma cells, osteoclasts, fibrosis, or sclerosis) with no bacterial growth while not on antibiotic therapy

Over the last 5 years, one of the authors (L.I.) has diagnosed nine cases of CRMO. Each case presents uniquely, and often families come with prolonged journeys to diagnosis. The goal of this report is to highlight three representative cases that help to familiarize community pediatricians with some common attributes of this rare and debilitating disease. All cases are presented after signed consent from patients and their guardians (if <18) for presentation of de-identified data. The study was further approved by an IRB protocol at Columbia University Medical Center (AAAS 3818).

## Case Presentations

### Case A

Patient A was an otherwise healthy 10-year-old girl who participated in competitive figure skating. She experienced insidious onset left knee pain, which was 8/10 in severity and only moderately improved with ibuprofen. Her family initially thought this could be related to skating and waited for it to disappear. However, the pain persisted, and she developed a limping gait. She had no fevers or rash, nor recent illnesses, travel, or tick exposure.

**Figure 1:**
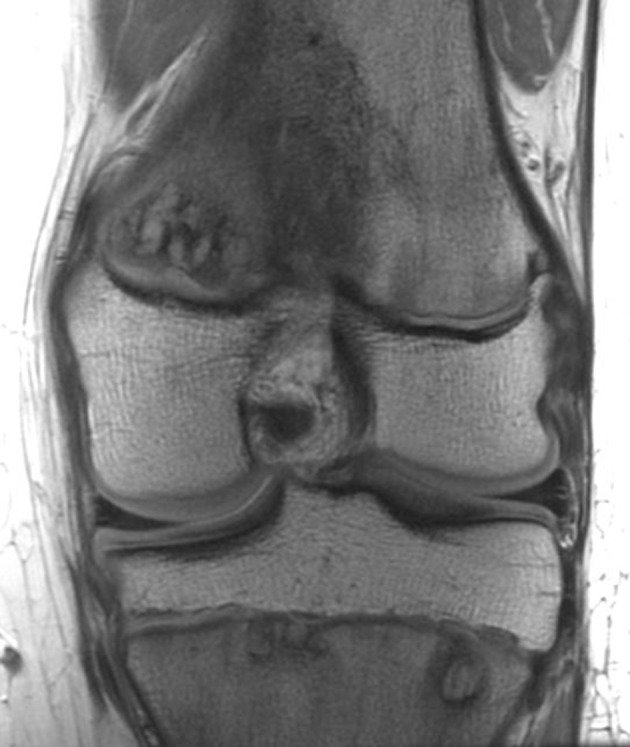
Multiple metaphyses affected in the left knee. Coronal proton density (MRI) image shows multiple areas of abnormality extending from the physes into the metaphyses of the distal femur and proximal tibia.

Her pediatrician referred her to orthopedics, who noted pain out of proportion to exam and ordered imaging. MRI of the left knee showed an intramedullary lesion in the proximal tibial and distal femoral metaphyses surrounded by edema and inflammation (Figure 1). Due to concern for potential malignancy, she was referred to oncology who ordered a technetium 99m-MDP bone scan and CT-guided biopsy. The bone scan was not consistent with acute osteomyelitis, with only mild radio-tracer uptake in the tibial lesion. Curiously, there was also a region of increased avidity in the posterior second rib. Subsequent tibial biopsy showed an inflammatory process in the marrow space extending to the growth plate. All cultures were negative.

The patient was referred to specialists in infectious disease whose differential diagnosis included acute bacterial osteomyelitis vs. CRMO. Due to elevated inflammatory markers, the patient was started on an empiric trial of antibiotics. Over the course of the next few months, her pain decreased, and her inflammatory markers and lesion on MRI imaging improved. The antibiotics were then discontinued after 4 months. Her knee pain remained quiescent for another 7 months but then recurred. Repeat MRI showed exacerbation of the tibial lesion, and repeat biopsy was again negative for growth of organisms. She was subsequently referred to our rheumatology practice for workup of CRMO (1 year after disease onset).

On initial visit, she had tenderness to palpation of the lateral proximal tibia. Her range of motion was fully intact, and inflammatory markers were, at this point, normal. WBMRI revealed lesions in eight regions, including her posterior rib (previously seen on bone scan), T9 vertebral body, bilateral sacroiliac joints, bilateral distal femoral metaphyses, and bilateral proximal tibial metaphyses. She was started on indomethacin, which provided some benefit but did not completely eliminate her pain. Methotrexate was added along with a brief course of oral prednisone, since glucocorticoids can help prevent vertebral collapse in the setting of spinal lesions (11, 12). She developed new bone lesions while on therapy and was switched to adalimumab (TNF-α inhibitor). She is not currently experiencing any of her pain and has returned to figure skating competition.

### Case B

Patient B was a 10-year-old girl with a history of scoliosis and hypothyroidism, whose family was living in Europe at the time of symptom onset. She was playing with a sibling and experienced significant pain when poked in the left clavicle. There was no fever or swelling, but the pain persisted, and her primary pediatrician referred her to orthopedics. Radiographs showed no evidence of fracture or dislocation, and she was prescribed ibuprofen. This did not provide relief, as she continued to have intermittent clavicular pain and occasional jaw pain.

She tolerated this discomfort into the next year when her endocrinologist decided to admit her for further workup. Lab evaluation showed elevated inflammatory markers, and she was given antibiotics, which did not lead to symptomatic improvement.

Her family moved back to the United States and visited a hospital, where they were told that she had chronic osteomyelitis and that it would resolve with time. They sought a second opinion at another hospital, where a bone biopsy was performed, which showed no evidence of infection. Her inflammatory markers were still elevated, and she was again trialed on antibiotics. As with before, this did not yield improvement, and they were discontinued due to gastrointestinal intolerance.

She then visited a third hospital, where a rheumatologist clarified her diagnosis as CRMO (3 years after disease onset). She was initially treated with indomethacin but discontinued it due to headaches. Over the next few years, she controlled her flares with ibuprofen. Eventually she established care with a new rheumatologist who started her on sulfasalazine, but this was discontinued.

Over time, her jaw pain worsened, and repeat MRI showed evidence of mandibular lesions. Etanercept (TNF-α inhibitor) was trialed and did not help, and after moving cities, her new pediatrician prescribed high dose steroids and referred her to our practice (7 years after disease onset).

**Figure 2:**
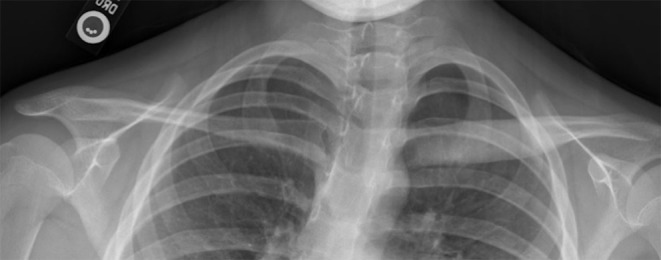
Involvement of the left clavicle. Radiograph obtained 2 years after disease onset showing that the left clavicle is broader and denser than the right, indicating chronic changes.

At the time of initial visit, she continued to complain of jaw pain and swelling. Physical exam was positive for jaw edema, and tenderness over the left clavicle (Figure 2). There was no loss in range of motion. WBMRI revealed an additional lesion in her right clavicle, and she was started on adalimumab, on which she has had improvement in inflammatory markers and pain level.

### Case C

Patient C was also a 10-year-old girl, who was otherwise healthy other than exhibiting poor growth. At baseline, she was involved in gymnastics; however, this changed when she started to experience right hip pain. This pain persisted at all times of day and was partially relieved by ibuprofen. It was never in the left hip or knees, but she reported some discomfort in her ankles. She had no fevers but developed a transient pustular rash on her palms and soles.

**Figure 3:**
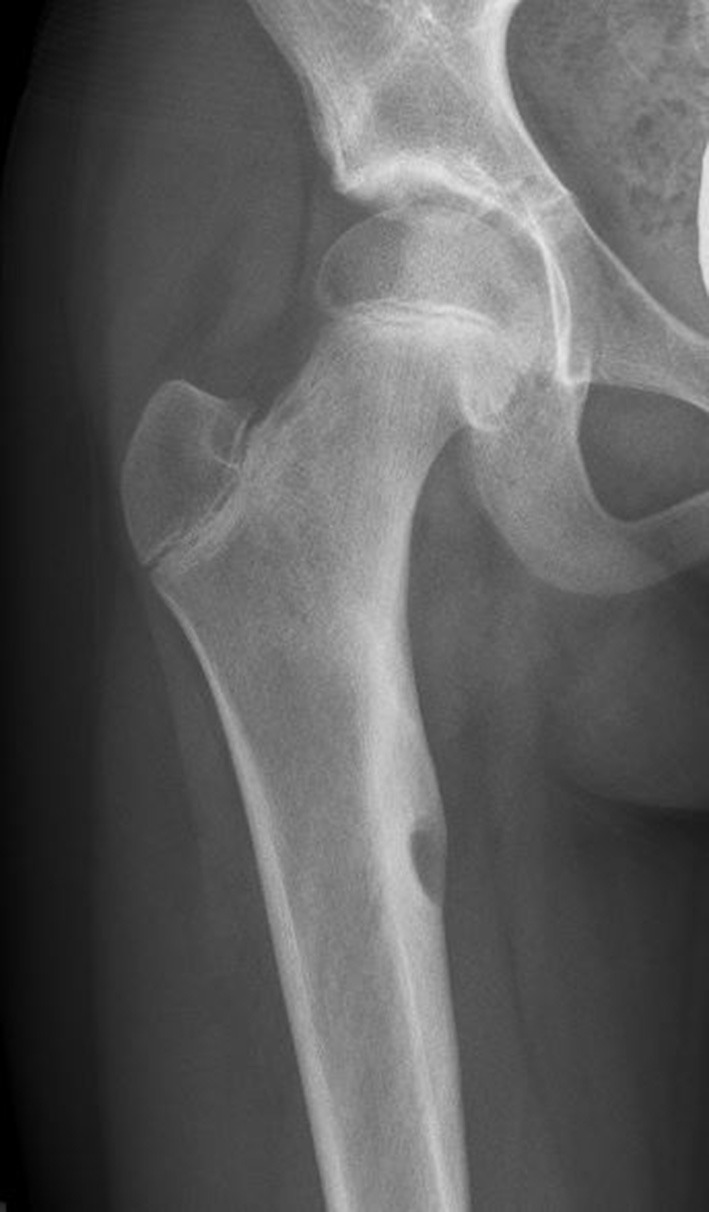
Involvement of the proximal right femur. Anterior-posterior radiograph shows a well-defined lucent lesion in the proximal diaphysis of the right femur with surrounding sclerosis, indicating chronic osseous destruction.

As the pain became increasingly disabling, she developed a limp. She was referred to orthopedics, who prescribed crutches and obtained a hip MRI. This showed a lesion in the right medial proximal femur (Figure 3). A bone scan was then performed to look for additional lesions, but none were found. Biopsy of the lesion was consistent with osteomyelitis but was undifferentiated between an infectious vs. inflammatory cause. The patient was thus referred to both infectious disease and rheumatology.

Infectious disease specialists suggested that the patient's presentation was more consistent with CRMO than bacterial osteomyelitis, given the chronicity and negative culture on biopsy. Our rheumatology team came to the same conclusion (1-year after symptom onset). When we saw the patient, she was tired and pale appearing, with thinning hair. Her BMI percentile was 1%. Musculoskeletal examination was notable for tenderness in the sacroiliac region and right femur, as well as reduced range of motion of her ankles. There were no skin lesions at the time of our exam. WBMRI showed lesions in her proximal right humerus, proximal right femur, bilateral proximal tibias, and bilateral distal femurs. She was treated with indomethacin and has since clinically improved.

Given her history of poor growth, a fecal calprotectin was performed to screen for IBD and was positive. This led to a colonoscopy, which showed granulomas throughout her intestinal tract (Chronic Granulomatous Disease was evaluated and excluded). She was given the diagnosis of Crohn's disease despite her lack of gastrointestinal symptoms. Her CRMO treatment was advanced to adalimumab, which treats both disease processes, and she has symptomatically improved.

## Discussion

The above cases are representations of how challenging it is to diagnose CRMO, often involving many specialists over the course of months to years. Our recounting of patient narratives does have limitations. By the time patients reach our rheumatology practice they have had a long workup, and we cannot corroborate all the details of their prior encounters with physicians at different hospitals. To shorten the time to diagnosis, we aim to describe some findings within our patients' histories that can raise the index of suspicion for CRMO and to describe the relevant literature.

All of our patients were 10-year-old females. This is more than coincidence, as CRMO is more likely to affect females than males (2:1), and most studies report a median age of onset around 10 or 11. All patients presented initially with one predominant area of pain, although there were hints that something else could be amiss.

Patient A had a very typical workup for CRMO including: local imaging, bone scan (hint of a 2nd rib lesion), and bone biopsy. The main differential diagnoses from the beginning were CRMO and bacterial osteomyelitis. As with patient B, she was trialed on empiric antibiotics in the setting of elevated inflammatory markers (ESR/CRP), even though her bone cultures were negative, and she had been afebrile.

In a case-matched study between bacterial osteomyelitis and CRMO (4), CRP was more characteristically elevated in bacterial osteomyelitis than CRMO (CRP 31 ± 50 vs. 9.8 ± 15.9 mg/dl, (mean ± SD), *p* = 0.008), and fever was present in 38% of cases vs. 12% (*p* = 0.003), respectively. Other reports have found elevated inflammatory markers in 40–70% of CRMO patients (1, 9). Thus, the presence of elevated inflammatory markers does not, alone, distinguish between infection or CRMO such as with Patients A and B.

The other potentially discriminating feature between bacterial and inflammatory osteomyelitis in these patients was their culture-negative biopsies. It is not surprising that these results were interpreted cautiously. *Staphylococcus aureus* and *Streptococcus pyogenes* are some of the most common bacterial pathogens in childhood osteomyelitis over the age of five. However, it is not uncommon for (presumed) infectious osteomyelitis to show no growth of organisms (13). Given infectious osteomyelitis is >20 times more common than CRMO and carries the potential for devastating sepsis if left untreated, many physicians will opt for empiric, broad-spectrum antibiotics if there is any ambiguity in diagnosis. While antibiotics can effectively treat culture-negative infectious osteomyelitis, they are less helpful for CRMO, which resolves with anti-inflammatory agents (14, 15). Thus, it is important to continue to follow these patients to ensure symptomatic resolution from their antibiotic course.

Patient A's clinical course was obscured by the fact that she did appear to improve after antibiotics. In retrospect, this may have reflected the relapsing and remitting course of CRMO symptoms. With a complaint as non-specific as knee pain, a diagnostic workup similar to Patient A may have been inevitable; however, in the setting of an afebrile, otherwise well-appearing patient, with chronic pain, hints on bone scan of more than one lesion, and a negative bone culture, CRMO should at least be on the differential (as it was for this patient).

The most salient feature of Patient B's history was her initial presentation with clavicular pain. Although the most common region for CRMO lesions is the long bones of the lower extremities, the clavicle is also characteristic, whereas it is an extremely uncommon location for bacterial osteomyelitis (4, 9). Patient B was eventually found to have bilateral clavicular lesions. While this finding came after diagnosis, it also reflects two unique features of CRMO—the presence of multifocal lesions as well as symmetric lesions (16).

Patient C's workup had several features suggestive of an underlying systemic inflammatory process. First, she had a history of poor growth. This led to a suspicion of IBD, as this condition is seen in ~10% of patients with CRMO. Despite her lack of overt symptoms, the bowel inflammation that was eventually diagnosed by endoscopy may have preceded her CRMO, explaining her failure to thrive (17). The other red flags in her history were her skin findings of palmoplantar pustulosis and her sacroiliac pain, findings seen in 8 and 25% of CRMO patients, respectively (2).

All patients underwent local imaging and biopsy during workup. Although the results do not always discriminate between infectious and inflammatory osteomyelitis, diagnosis of CRMO can often be confirmed if whole body imaging is performed. In light of this, one approach may be to perform WBMRI at an earlier time point. There is currently no algorithm for when such a study is likely to have the highest yield and cost-effectiveness based on a pre-test probability; however, there are potentially unique local imaging features that could help, which is currently under investigation by our team.

As another possible solution, other groups have been evaluating novel laboratory measures, since white blood cell count and ESR/CRP are non-specific. A study from 2013 divided patients into: (1) confirmed pyogenic, (2) presumed pyogenic, and (3) inflammatory osteomyelitis/arthritis. A serum procalcitonin threshold of 0.4 ng/mL had a 85.2% sensitivity and 87.3% specificity in discriminating groups 1+2 from group 3, suggesting that procalcitonin could be a useful diagnostic tool to diagnose infectious vs. inflammatory osteomyelitis (18).

Once diagnosed, first line treatment involves NSAIDS, particularly indomethacin and naproxen. On such a regimen, >50% of patients will experience clinical improvement within 12 months, as described in detail by Beck et al. (19). The Childhood Arthritis and Rheumatology Research Alliance (CARRA) has proposed 3 second line strategies: (1) methotrexate or sulfasalazine, (2) TNF-α inhibitors ± methotrexate, and (3) bisphosphonates (12). These can be used in addition to NSAIDS or can replace them if patients cannot tolerate the gastrointestinal side effects. Finally, bisphosphonates or short courses of steroids should be considered for patients with spinal lesions who are at risk of vertebral collapse.

## Conclusion

In summary, CRMO is an autoinflammatory disease of the bone in children that can cause chronic debilitating pain and disability. CRMO has onset around age 10 and affects girls more than boys in a 2:1 ratio. While rare, it is likely underdiagnosed, and better awareness may help reveal missed diagnoses and expedite care. Clues toward CRMO from patient histories and workup include: chronic bone pain, culture negative biopsies, clavicular pain, and systemic inflammatory features such as IBD. Conversely, if a diagnosis of CRMO has been made, it is important to screen for these other features, as IBD can be detrimental to growth and development. While children may have different medication tolerances, most do well on NSAIDs, methotrexate, and/or TNF-α inhibitors. Systemic steroids are not usually necessary but may be indicated if the lesions involve vertebral bodies or have caused a bone fracture. The natural history for most patients is for the disease to be self-limited.

## Ethics Statement

The studies involving human participants were reviewed and approved by an IRB protocol at Columbia University Medical Center (AAAS 3818). Written informed consent to participate in this study was provided by the participants' legal guardian/next of kin.

## Author Contributions

All authors listed have made a substantial, direct and intellectual contribution to the work, and approved it for publication.

### Conflict of Interest

The authors declare that the research was conducted in the absence of any commercial or financial relationships that could be construed as a potential conflict of interest.
